# Bowing Gestures Classification in Violin Performance: A Machine Learning Approach

**DOI:** 10.3389/fpsyg.2019.00344

**Published:** 2019-03-04

**Authors:** David Dalmazzo, Rafael Ramírez

**Affiliations:** Music Technology Group, Department of Information and Communication Technologies, Universitat Pompeu Fabra, Barcelona, Spain

**Keywords:** machine learning, technology enhanced learning, Hidden Markov Model, IMU, bracelet, audio descriptors, bow strokes, sensors

## Abstract

Gestures in music are of paramount importance partly because they are directly linked to musicians' sound and expressiveness. At the same time, current motion capture technologies are capable of detecting body motion/gestures details very accurately. We present a machine learning approach to automatic violin bow gesture classification based on Hierarchical Hidden Markov Models (HHMM) and motion data. We recorded motion and audio data corresponding to seven representative bow techniques (*Détaché, Martelé, Spiccato, Ricochet, Sautillé, Staccato*, and *Bariolage*) performed by a professional violin player. We used the commercial *Myo* device for recording inertial motion information from the right forearm and synchronized it with audio recordings. Data was uploaded into an online public repository. After extracting features from both the motion and audio data, we trained an HHMM to identify the different bowing techniques automatically. Our model can determine the studied bowing techniques with over 94% accuracy. The results make feasible the application of this work in a practical learning scenario, where violin students can benefit from the real-time feedback provided by the system.

## 1. Introduction

A *gesture* is usually defined as a form of non-verbal communication action associated with an intention or an articulation of an emotional state. It constitutes an intrinsic part of the human language as a natural body-language execution. Armstrong et al. (1995) defined gestures as an underlying brain mechanism common in both language and motor functions. Gestures have been studied in the context of dance performance, sports, rehabilitation and music education, where the term is not only related to speech but is interpreted as the broader concept of a “*learned technique of the body”* (Carrie, 2009). For instance, in highly competitive sports, as well as in music education, gestures are assumed to be automatic-motor abilities, learned by repetition, to execute an action optimally. Therefore, those gestures are intended to be part of the performer's repertoire. Gestures in music are of paramount importance because fine postural and gestural body movements are directly linked to musicians' expressive capabilities, and they can be understood as well as correct “*energy-consumption”* habit development to avoid injuries.

Current motion capture technologies are capable of detecting body motion details very accurately, and they have been used in a variety of sports industries to enhance athletes throughput, or in rehabilitation applications (Chi et al., 2005). For instance, tracking systems have been built into professional golf clubs as a computer assistant to strengthen swing analysis. Bilodeau et al. (1959) argue that real-time feedback has a more positive effect on learning new motor skills. Furthermore, in musical education, implementing similar computer-assisted methodologies, tracking systems and inertial measurement units (IMU) are recently being developed with the aim to improve music education, instruction and performance. 3D body reconstructions based on camera motion tracking rooms or electromagnetic positional tracking systems can be quite expensive. Hence, new models using wearable devices based on magnetometers, gyroscopes and accelerometers, in conjunction with machine learning algorithms are being reported as efficient and low-cost solutions for analyzing body motion and gestural information (Mitra and Acharya, 2007). From this perspective, the Internet of Musical Things (IoMusT) is an emerging field as an extension of the Internet of Things principle. It refers to the design and implementation of embedded technology in smart music-instruments to expand its possibilities, recollect data from the users and enhance the learning process in particular of those cases of self-practice learners who do not have direct feedback from the tutor. Also, it fosters the design of new collaborative learning environments connected to an online application. The field of IoMusT embrace topics such as human-computer interaction, artificial intelligence, new interfaces for musical expression and performative arts (Turchet et al., 2017, 2018).

### 1.1. Motivation

TELMI (Technology Enhanced Learning of Musical Instrument Performance), is the framework where this study is being developed (TELMI, 2018). Its purpose is to investigate how technology regarding multimodal recordings, computer systems, sensors and software, can enhance music students practices, helping them to focus on the precise development of good habits, especially at the moment to incorporate better musical skills. With the focus on violin performance, as a test case, one of the primary goals of the project is to provide real-time feedback to students about their performance in comparison to good-practice models which are based on recordings of experts. Our findings would be implemented in other instruments in music education environments. Academically, the project is a collaboration between the Universitat Pompeu Fabra, the University of Genova and the Royal College of Music, London.

## 2. Related Work

### 2.1. Automatic Gesture Recognition

Among many existing machine learning algorithms, Hidden Markov models (HMMs) have been widely applied to motion and gesture recognition. HMMs describe motion-temporal *signature* events with internal discrete probabilistic states defined by Gaussian progressions (Brand et al., 1997; Wilson and Bobick, 1999; Bevilacqua et al., 2010; Caramiaux and Tanaka, 2013). They have been applied to music education, interactive installations, live performances, and studies in non-verbal motion communication. Yamato et al. (1992) is probably the first reference of applying HMMs to describe temporal events in consecutive-image sequences. The resulting model identified with high accuracy (around 90%) six different tennis stroke gestures. Brand et al. (1997) presented a method based on two coupled HMMs as a suitable strategy for highly accurate action recognition and description over discrete temporal events. In their study, they defined T'ai Chi gestures tracked by a set of two cameras, in which a blob is extracted forming a 3D model of the hand's centroids. Authors argued that simple HMMs were not accurate enough where coupled HMMs succeed in classification and regression. Wilson and Bobick (1999) introduced an on-line algorithm for learning and classifying gestural postures in the context of interactive interfaces design. The authors applied computer vision techniques to extract body and hands positions from camera information and defined an HMM with a structure based on Markov Chains to identify when a gesture is being performed without previous training. In another study conducted by Yoon et al. (2001), an HMM is used to develop a hand tracking, hand location and gesture identification system based on computer vision techniques. Based on a database consisting of hand positions, velocity and angles, it employs k-means clustering together with an HMM, to accurately classify 2,400 hand gestures. The resulting system can control a graphical editor consisting of twelve 2D primitives shapes (lines, rectangles, triangles, etc.) and 36 alphanumeric characters. Haker et al. (2009) have presented a detector of deictic gestures based on a time-of-flight (TOF) camera. The model can determine if a gesture is related to the specific meaning by pointing to some information projected in a board; it also handles a slide-show, switching to the previous or next slide with a thumb-index finger gesture. Kerber et al. (2017) presented a method based on a support vector machine (SVM) in a custom Python program, to recognize 40 gestures in real-time. Gestures are defined by finger dispositions and hand orientations. Motion data is acquired using the *Myo* device with an overall accuracy of 95% of correct gestures estimations. Authors have implemented a matrix automatic transposition allowing the user to place the armband with any precise alignment or left/right forearm considerations.

### 2.2. Automatic Music Gesture Recognition

There have been several approaches to study gestures in a musical context. Sawada and Hashimoto (1997) applied an IMU device consisting of an accelerometer sensor to describe rotational and directional attributes to classify music gestural expressions. Their motivation was to measure non-verbal communication and emotional intentions in music performance. They applied tempo recognition in orchestra conductors to describe how the gestural information is imprinted in the musical outcome. Peiper et al. (2003) presented a study of violin bow articulations classification. They applied a decision tree algorithm to identify four standard bow articulations, *Détaché, Martelé, Spiccato*, and *Staccato*. The gestural information is extracted using an electromagnetic motion tracking device mounted close to the performer right hand. The visual outcome is displayed in a CAVE as a room with a four-wall projection setup for immersive virtual reality applications and research. Their system reported high accuracy (around 85%) when classifying two gestures; however, the accuracy decreased to 71% when four or more articulations were considered.

Kolesnik and Wanderley (2005) implemented a discrete Hidden Markov Model for gestural timing recognition and applied it to perform and generate musical or gestural related sounds. Their model is able to be trained with arbitrary gestures to track the user's motion. Gibet et al. (2005) developed an “augmented violin” as an acoustic instrument with aggregated gestural electronic-sound manipulation. They modeled a k-Nearest Neighbor (k-NN) algorithm for the classification of three standard violin bow strokes: Détaché, Martelé and Spiccato. Authors used an analog device (i.e., ADXL202), placed at the bow-frog, to transmit bow inertial motion information. It consisted of two accelerometers to detect bowing direction. Gibet et al. (2005) described a linear discrete analysis to identify important spacial dissimilitudes among bow articulations, giving a highly accurate gestural prediction in the three models presented (Detaché 96.7%, Martelé 85.8%, and Spiccato 89.0%). They also described a k-NN model with 100% accuracy estimation in Detaché and Martelé, 68.7% in Spiccato. They conclude that accuracy is directly related to dynamics, i.e., *pp, mf* and *ff*. Caramiaux et al. (2009) presented a real-time gesture follower and recognition model based on HMMs. The system was applied to music education, music performances, dance performances, and interactive installations. Vatavu et al. (2009) proposed a naive detection algorithm to discretize temporal events in a two-dimensional gestural drawing matrix. The similitude between two gestures (template vs. new drawing) is computed with a minimum alignment cost between the curvature functions of both gestures. Bianco et al. (2009) addressed the question of how to describe acoustic sound variations directly mapped to gesture performance articulations based on the Principal component analysis (PCA) and segmentation in their sound analysis. They focused on the study of a professional trumpet player performing a set of exercises with specific dynamical changes. The authors claimed that the relationship between gestures and sound is not linear, hypothesizing the at least two motor-cortex control events are involved in the performance of single notes.

Caramiaux et al. (2009) presented a method called canonical correlation analysis (CCA) as a gesture tool to describe the relationship among sound and its corresponding motion-gestural actions in musical performance. The study is based on the principle that speech and gestures are complementary and co-expressive in human communication. Also, imagery-speech can be reported as a muscular activity in the mandibular area. The study described features extracted to define motion in body movements, defining a multi-dimensional stream with coordinates, vector velocities and acceleration to represent a trajectory over time; as well as its correlation with sound features, giving an insight on methodologies to extract useful information and describe sound-gesture relationships. Tuuri (2009) proposed a gestural-based model as an interface for sound design. Following the principle that stereotypical gesture expression communicates intentions and represent non-linguistic meanings, sound can be modeled as an extension of the dynamical changes naturally involved on those gestures. In his study, he described body movement as *semantics* regarding sound design.

Bevilacqua et al. (2010) presented a study in which an HMM-based system is implemented. Their goal was not to describe a specific gestural repertoire, but instead, they proposed an optimal “*low-cost”* algorithm for any gestural classification without the need for big datasets. Gillian et al. (2011) exposed a different approach to the standard Markov Model described above. They extended Dynamic Time Warping (DTW) to classify N-dimensional signal with a low number of training samples, having an accuracy rate of 99%. To test DTW algorithms, the authors first defined a set of 10 gestures as an “*air drawing”* articulations of the right hand. Drawn numbers from 1 to 5, a square, a circle, a triangle, a horizontal and vertical gestural line similar to an orchestral conducting, were the final gestural repertoire. Their methodology, in conclusion, gives a valid and optimal approach to classify any gesture. In the same year, a study conducted by Van Der Linden et al. (2011) described the invention of a set of sensors and wearables called *MusicJacket*. They aimed to give postural feedback and bowing technique references to novice violin players. Authors reported that vibrotactile feedback directly engages the subjects' motor learning systems, correcting their postures almost immediately, shortening the period needed to acquire motor skills and reduces cognitive overload.

Schedel and Fiebrink (2011), have implemented the Wekinator application Fiebrink and Cook (2010) to classify seven standard cello bow articulations such as *legato, spiccato*, or *marcato*, among others. Using a commercial IMU device known as K-Bow for the motion data acquisition. The cello performer used a foot pedal to stop and start articulation training examples. For each stroke, she varied the string, bow position, bow pressure, and bow speed. After training a model, the cellist evaluated it by demonstrating different articulations. The authors created an interactive system for composition and sound manipulation in real-time based on the bow gesture classifications. Françoise et al. (2014) introduced the “*mapping by demonstration”* principle where users create their gestural repertoire by simple-direct examples in real-time. Françoise et al. (2012, 2014) presented a set of probabilistic models [i.e., Gaussian Mixture Models (GMM), Gaussian Mixture Regression (GMR), Hierarchical HMM (HHMM) and Multimodal Hierarchical HMM (MHMM), Schnell et al., 2009] and compared their features for real-time sound mapping manipulation.

In the context of IoMusT, Turchet et al. (2018b) extended a percussive instrument called *Cajón* with embedded technology such as piezo pickups, condenser microphone and a Beaglebone Black board audio processor with WIFI connectivity. Authors have applied machine learning (k-NN) and real-time onset detection techniques to classify the hit-locations, dynamics and gestural timbres of professional performers with accuracies over 90% on timber estimations and 100% on onset and hit location detection.

## 3. Materials and Methods

### 3.1. Music Materials

**Figure 1:**
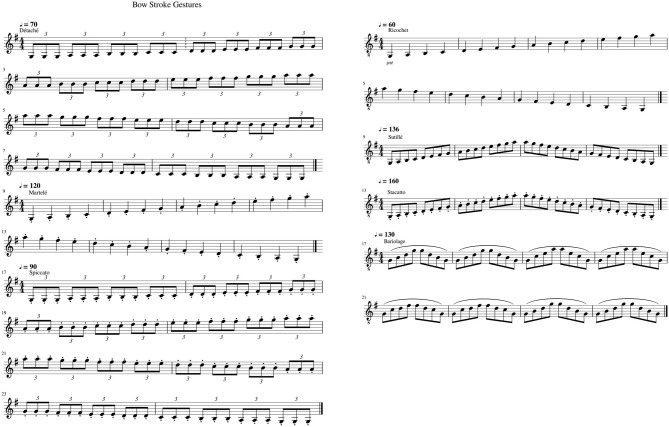
Music score of the seven bow strokes. All in G Mayor as explained in Music Material section.

In collaboration with the Royal College of Music, London, a set of seven gestural violin-bowing techniques were recorded as a reference by professional violinist Madeleine Mitchell. All gestures were played in G mayor for technical accommodation to cover three octaves using a comprehensive violin range within the four strings. Below we describe the seven recorded bowing gestures (music score reference in Figure 1):

- ***Détaché*.** It means *separated*; the method describes a clean, stable sound with each bowing direction, moving smoothly from one note to the next. The weight over the violin strings is even for each note performed. It is the most common bowing technique in the violin repertoire. The exercise was performed within two octaves ascending and descending scale in 4/4 at 70BPM, playing three eighth-triplet per note. In total 32 bow-strokes samples were recorded.
- ***Martelé*.** The term means *hammered*; it is an extension of *Détaché*. with a more distinctive attack, caused by a faster and slightly stronger initial movement to emphasize the motion starting point and it has a moment of silence at the end. Two octaves were played at 120 BPM in 4/4 played with Quarter-notes. 32 bow-stroke samples were recorded in total.
- ***Spiccato*.** It is a light bouncing of the bow against the strings. It is achieved by the physical effect of attacking the strings on a vertical (horizontal) angular approach of the bow with a controlled weight and a precise hand-wrist control. Two octaves performed at 90 BPM attacking each note with three eighth-triplets. 32 bow-stroke samples were recorded in total.
- ***Ricochet*.** Also known as *Jeté*, it is a controlled bouncing effect played in a single down-bowed stroke starting with a *Staccato* attack but controlling the weight of the Bow against the Violin's string with the wrist. The bouncing produces a rhythmic pattern, usually, among two to six notes. In this particular example, three eight notes (triplet) where produced for each bow-stroke notated as a quarter note in the musical score. Two octaves were played at 60 BPM in 4/4.
- ***Sautillé*.** This technique implies fast notes played using one bow-stroke per note. The bow bounces slightly over the string, and the hair of the bow retains some slight contact with it. Two octaves were played at 136 BPM in 4/4 with eighth-notes rhythmic pattern per note of the scale. 32 bow-stroke samples were recorded in total.
- ***Staccato*.** A similar gesture to *Martelé*. It is a clean attack generated by a controlled pressure over the string with an accentuated released in the direction of the bow-stroke. It is controlled by a slight rotation of the forearm where pronation attacks the sound and supination released it; it can be generated by up and down motion of the wrist, or a pinched gesture with the index finger and the thumb. Two octaves were played at 160 BPM in 4/4, quarter-notes to generate each note. We estimated four groups of notes as part of the gesture having in total eight gestures.
- ***Bariolage*.** It means *multi-colored*, to express an ascending or descending musical phrase. It is the bowing technique to cover a group of changing notes in one bow-stroke direction usually in adjacent strings. Eight arpeggios where played at 130BPM in 4/4 in a rhythmic pattern of eight-notes, each one played two times drawing the harmony progression of I–ii2–Vsus4–I.

In total 8,020 samples within seven gestures, with a median of 35.8 samples per bow-stroke, having 32 bow-strokes per gesture. Each bow-stroke covers a time window range approximately of 200 ms.

### 3.2. Data Acquisition, Synchronization, and Processing

- ***Myo*** A highly sensitive nine-axis IMU device Myo was used to acquire information from the right forearm-motion during the gesture recordings. *Myo* is a bracelet composed of a set of sensors for motion estimation and a haptic feedback motor. The bracelet size is between 19 and 34 cm adjustable to the forearm circumference. It weighs 93 grams. The hardware is composed of eight medical grade stainless steel EMG sensors that report electrical muscle activity. The IMU contains three-axis gyroscope giving degrees of change in radians per second (angular velocity), three-axis accelerometer as an estimation of -8g to 8g (1g=9.81 *m*/*s*^2^), three-axis magnetometer giving as an output a Quaternion reference of the imaginary rotation of the *Myo* in the space. It has an ARM Cortex M4 Processor, and it may provide short, medium and long haptic feedback vibration. Its communication with a computer is based on Bluetooth with an included adapter, giving a sampling rate of 200Hz (Hop-time of 5 ms).
- ***Openframeworks (OF)*** (c++ open-source framework, 2018) was used to acquire, visualize the IMU's information in real-time and play the audio files in synchronization with the Myo device. OF is an open-source platform based on C++ which has a collection of libraries to develop applications in all operating systems. Developers and artists commonly use it in the field of interactive applications, video games, and mobile apps. We have developed an additional library to receive *Myo's* information which is released as an OF *Addon*^1^ Our library translates Myo's motion data into a compatible OF format and makes CSV databases for motion analysis.
- ***Max/MSP*** is a visual programming language platform commonly used in electronic music and interactive media development and creation, suitable for quick prototyping and it allows communication with external devices.
- ***Essentia*** is an Open-source C++ library and tools for audio and music analysis, description and synthesis. It is developed in MTG-Pompeu Fabra University (http://essentia.upf.edu). *Essentia* has many different algorithms that can be custom designed. Using the standard setup list of values as acoustic characteristics of the sound is computed, producing spectral, temporal, tonal or rhythmic descriptors. Essentia is included in the custom application using *ofxAudioAnalyzer* (Leozimmerman, 2017).
- **Synchronization** The synchronization of the multimodal data is divided into two phases.
  **Recording**: At the moment to record the gestures and synchronize the *Myo* device with video and audio data, we implemented a *Max/MSP* program which sends OSC events to the *Myo* application to generate a database of CSV files, it records the data at 60 fps. These files are created taking into account a synchronization format: timer in milliseconds, accelerometer (x, y, z), gyroscope (x, y, z), Quaternion (w,x,y,z), electromyogram (eight values), point_vector(x,y,z), point_direction (x,y,z), point_velocity(x,y,z), event (it is a marker during recordings). Those CSV files are recorded in the same time-window range reference of the audio data, also created within Max. The format of the video, myo and audio files are defined by counter_gesture_second-minute-hour_day-month-year (extension are .csv, .mov or .wav), where the counter is the iteration of the recording session, the gesture is the identificator number and time/date description to pair all files and avoid overwriting. The master recorder in Max/MSP sends the global timer (ms) reference to the Myo application which is reported in the CSV file. To acquire audio we used an interface Zoom H5 linked to Max, recording WAV files with a sample rate of 44.100 Hz/16 bits. *Myo* device running in a MacBook Pro (13-inch, 2017), 2.5 GHz Intel Core i7 processor and a memory of 8 GB 2133 MHz LPDDR3 with a latency of 10ms to 15ms. However, the final alignment is controlled by the Openframeworks app. The sound file reader reports the millisecond where the audio is being read, then, that value is passed to the CSV reader with an offset of -10 (ms) giving the motion information to be visualized.
  **Testing**: Data from the Myo application is sent to Max/MSP to train and test the machine learning models. This data is an OSC message bundle which consists of a Timer, Euler Angles (x,y,z), Gyroscope (x,y,z), Accelerometer (x,y,z), RMS, Pitch Confidence, Bow-Stroke (reference of the gesture-sample) and Class (gesture identificator). (Essentia features are explained in section Audio Analysis) The application runs at 60 fps, where the Essentia setup is: sample rate of 44,100 Hz, a buffer of 512 samples, two channels (stereo), having a latency of 12ms. OSC package sent from OF application to MAX/MSP, reads the Myo data and obtains the audio descriptors (Essentia) in a process that takes one cycle (16.6666 ms) and any time alignments or offset between both sources is considered.

### 3.3. Methods

#### 3.3.1. Audio analysis

The Essentia library was used to extract audio features from the recordings. The descriptors extracted with real-time audio buffering analysis were:

- RMS: The Root-Mean-Square descriptor informs about the absolute area under the audio waveform. In other words, it describes the power voltage that the waveform sends to the amplifier.
- Onset: It is a normalized value (0.0 to 1.0) which reports locations within the frame in which the onset of a musical phrase, rhythm (percussive event) or note has occurred.
- Pitch Confidence: It is a range value from zero to one to determine how stable the description of a pitch presence is in a defined windowing buffer as opposed to non-harmonic or not tonally defined sound.
- Pitch Salience: It is a measure of tone sensation, which describes in a range from zero to one when a sound contains several harmonics in its spectrum. It may be useful to discriminate, for instance, between rhythmic sound presence and instrumental pitched sound presence.
- Spectral Complexity: It is based on the number of peaks in the sound spectrum referred to a windowing sound buffer. It is defined as the ratio between the spectrum's maximum peak's magnitude and the “bandwidth” of the peak above half its amplitude. This ratio reveals whether the spectrum presents a pronounced maximum peak.
- Strong Decay: A normalized value that gives a reference to express how strong or pronounced is the distance between the sound power centroid to its attack. Hence, a signal containing a temporal centroid near its start boundary and high energy is said to have a steady decay.

We used RMS, Pitch Confidence and Onset to segment the Myo gesture to eliminate non-gesture data. In this way, we defined meaningful gesture time-intervals and used the corresponding Myo data for training the system.

Also, to use audio descriptors for data segmentation, a second objective was to complement the Myo information with relevant audio information to train the machine learning models with multimodal data. While the *Myo* provides information about forearm motion, it does not directly report activity of the performer fine-movements from the wrist and fingers; the audio analysis may provide information relevant to those gestural characteristics. A custom application build on Openframeworks was used to read in real-time the data from the *Myo*, record CSV files with events, synchronize with the audio recordings, and read the synchronized data from the motion files and audio files, automatically. It is also possible to automatically send the data to Repovizz, an on-line publicly available repository (Mayor et al., 2011).

### 3.4. Classification Models

**Figure 2:**
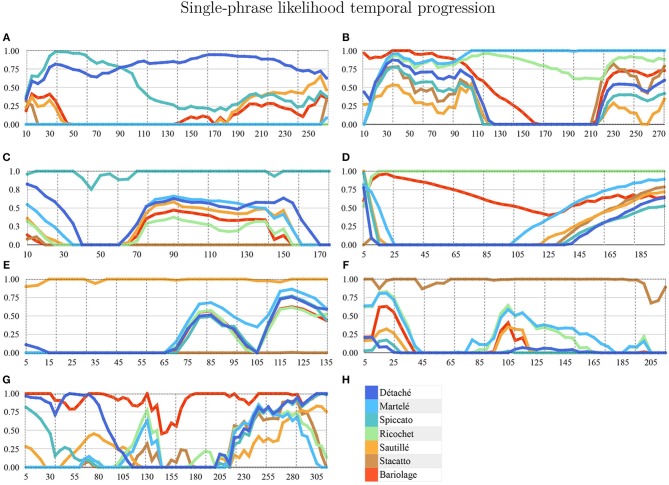
HHMM-likelihood progression of a single bow-stroke phrase example in each technique. x-axis is time (ms) and y-axis is percentage of correct prediction (1:100). **(A)** Détaché, **(B)** Martelé, **(C)** Spiccato, **(D)** Ricochet, **(E)** Sautillé, **(F)** Staccato, **(G)** Bariolage, and **(H)** Color-label per bow stroke.

We applied a Hierarchical Hidden Markov Model (HHMM) for real-time continuous gesture recognition (Schnell et al., 2009). We built three different gestural phases of the violin bow strokes and defined a model with ten states. States are used for segmentation of the temporal windowing of each bow stroke. The model provides a probabilistic estimation of the gesture being performed. Hence, those ten states are composed of ten Gaussian mixture components, which reports out the likelihood estimation on a scale from 0. to 1.0. We used a regularization in a range of [0.01, 0.001] to filter noise. In Figure 2 all bow-strokes are taken randomly to visualize the probabilistic output of the likelihood, for instance in the first bow-stroke (*Détaché*), the first three likelihood progression reported *Martelé*.

Three different musical phrases covering low, mid and high pitch registers were provided for each gesture as performed by the expert. Hence, the model was trained using examples of “*good practice”*, following the principle of mapping by demonstration (Françoise et al., 2012).

**Figure 3:**
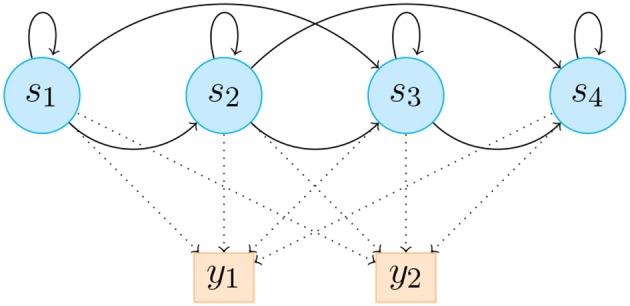
HHMM illustration consists of 4 states, which emit 2 discrete likelihood estimations *y*_1_ and *y*_2_. *a*_*ij*_ is the probability to transition from state *s*_*i*_ to state *s*_*j*_, and *b*_*j*_(*y*_*k*_) is the probability to emit likelihood *y*_*k*_ in state *s*_*j*_. Solid lines represent state transition probabilities *a*_*ij*_ and dotted lines represent *b*_*j*_(*y*_*k*_).

**Figure 4:**
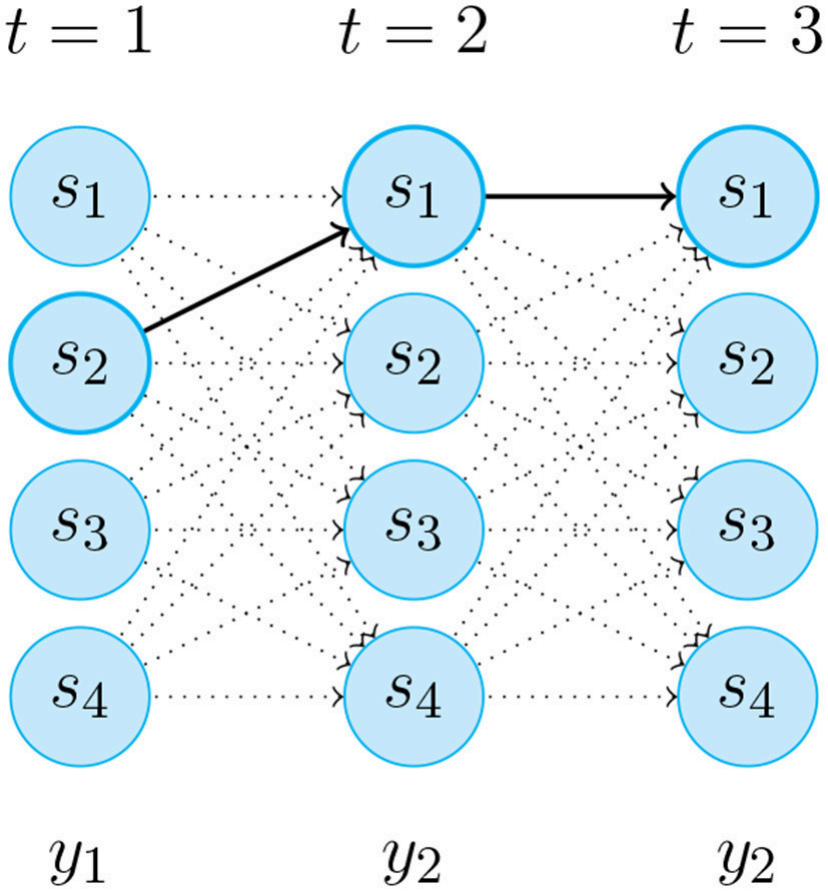
An instance of the *Likelihood* progression fulfillment of the unobserved Markov chain sequence *y*_1_,*y*_2_,*y*_2_ for HMM in Figure 2. The thick arrows indicate the most probable transitions.

Following this methodology, it is possible to have accurate results without the need for a big dataset of training examples. The data is sent from the custom application to the Max implementation through OSC (explained in Synchronization section). For the regression phase, the HHMM provides an output with a normalized number corresponding to the gesture prediction, and a set of values called *likelihood* as a temporal description of the Gaussian probability distribution in time, covering the ten following states of the bow stroke (Figures 2–4).

We evaluated three HHMMs: one trained with the information from the *Myo* sensors, a second model was trained with the audio descriptors previously described, and a third model trained with a selection of both, motion and audio descriptors. Table 1 shows the complete descriptors included in each motion, audio and combined datasets. Applying automatic feature selection algorithms in WEKA, we have finally discarded some of the audio descriptors (Onset, Pitch Salience, Spectral Complexity, Strong Decay) that were reported as not strongly informative to the gestures quality.

**Table 1:** Databases setup.

**Dataset**	**Features**
Audio	RMS, Onset, Pitch Confidence, Pitch Salience, Spectral Complexity, Strong Decay
Myo (IMU)	Euler, Accelerometer, Gyroscope
Combined Audio and Myo	Euler, Accelerometer, Gyroscope, RMS, Pitch Confidence

## 4. Results

We trained decision trees models using three feature sets: Myo motion features, audio features, and motion and audio features combined. Applying 10-fold cross-validation, we obtained correctly classified instances percentages of 93.32, 39.01, and 94.62% for the motion only, audio only, and combined feature sets, respectively. As it can be seen in the confusion matrix reported in Table 2, we obtained an accuracy per gesture of (a) 96.3%, (b) 95%, (c) 99.9%, (d) 95.1%, (e) 95.5%, (f) 72.5%, (g) 88.2% for detache, martele, spiccato, ricochet, sautille, Staccato, and bariologe, respectively. Table 3 gives the detailed statistics for each gesture. In addition, we trained an HHMM with the combined motion and audio dataset. In the reminder of the paper, we will report the results of this model.

**Table 2:** Confusion matrix (decision tree).

**a**	**b**	**c**	**d**	**e**	**f**	**g**	**Class**
**0.963**	0.000	0.005	0.001	0.031	0.000	0.000	**a**
0.001	**0.950**	0.000	0.027	0.000	0.011	0.012	**b**
0.000	0.001	**0.999**	0.000	0.000	0.000	0.000	**c**
0.000	0.025	0.001	**0.951**	0.000	0.017	0.006	**d**
0.040	0.002	0.000	0.001	**0.955**	0.001	0.000	**e**
0.000	0.092	0.000	0.095	0.003	**0.725**	0.084	**f**
0.000	0.030	0.000	0.037	0.000	0.050	**0.882**	**g**

*Confusion Matrix of Decision Tree Algorithm Blue numbers are the correct predicted gestures as percentages of correct trials. Values are scaled from 0 to 1. Letters correspond to: (a) Détaché, (b) Martelé, (c) Spiccato, (d) Ricochet, (e) Sautillé, (f) Staccato, and (g) Bariolage*.

**Table 3:** Accuracy by class (combined audio and motion).

**Class**	**TP rate**	**FP rate**	**Precision**	**Recall**	**F-Measure**	**MCC**	**ROC area**	**PRC area**
Détaché	0.963	0.005	0.979	0.963	0.971	0.964	0.988	0.967
Martelé	0.950	0.015	0.948	0.950	0.949	0.934	0.975	0.940
Spiccato	0.999	0.001	0.993	0.999	0.996	0.995	0.999	0.993
Ricochet	0.951	0.016	0.936	0.951	0.943	0.929	0.975	0.905
Sautillé	0.955	0.007	0.938	0.955	0.947	0.940	0.987	0.942
Staccato	0.725	0.010	0.773	0.725	0.749	0.738	0.903	0.682
Bariolage	0.882	0.008	0.889	0.882	0.886	0.877	0.960	0.865
Weighted Avg.	0.946	0.010	0.946	0.946	0.946	0.937	0.978	0.930

*TP Rate, True Positive Rate; FP Rate, False Positive Rate; MCC, Matthews Correlation Coefficient; ROC, Receiver Operating Characteristic; PRC, Precision-recall. Correctly Classified 94.625%. Incorrectly Classified 5.37%*.

**Figure 5:**
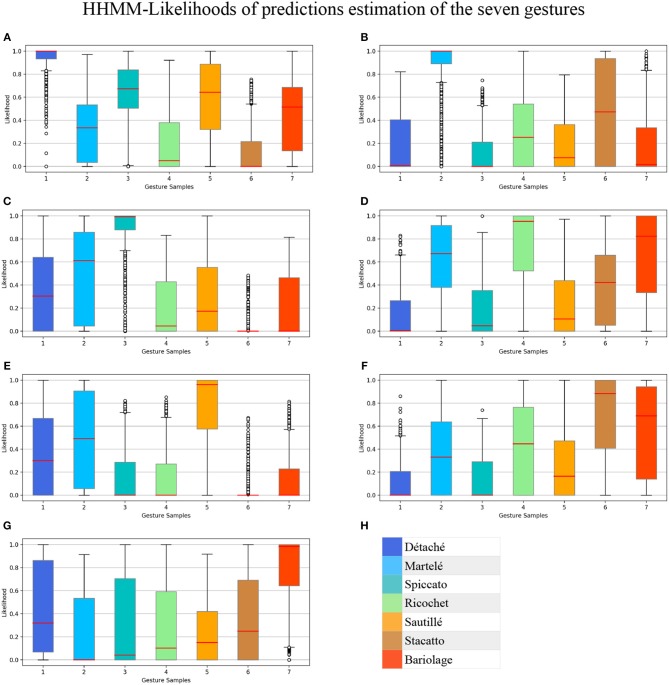
Box-plot summarizing all HHMM-likelihood progression in 7,846 samples with a mean of 42,394 samples per gesture. Bow strokes are organized as: **(A)** Détaché, **(B)** Martelé, **(C)** Spiccato, **(D)** Ricochet, **(E)** Sautillé, **(F)** Staccato, **(G)** Bariolage, and **(H)** Color-label per gesture.

**Figure 6:**
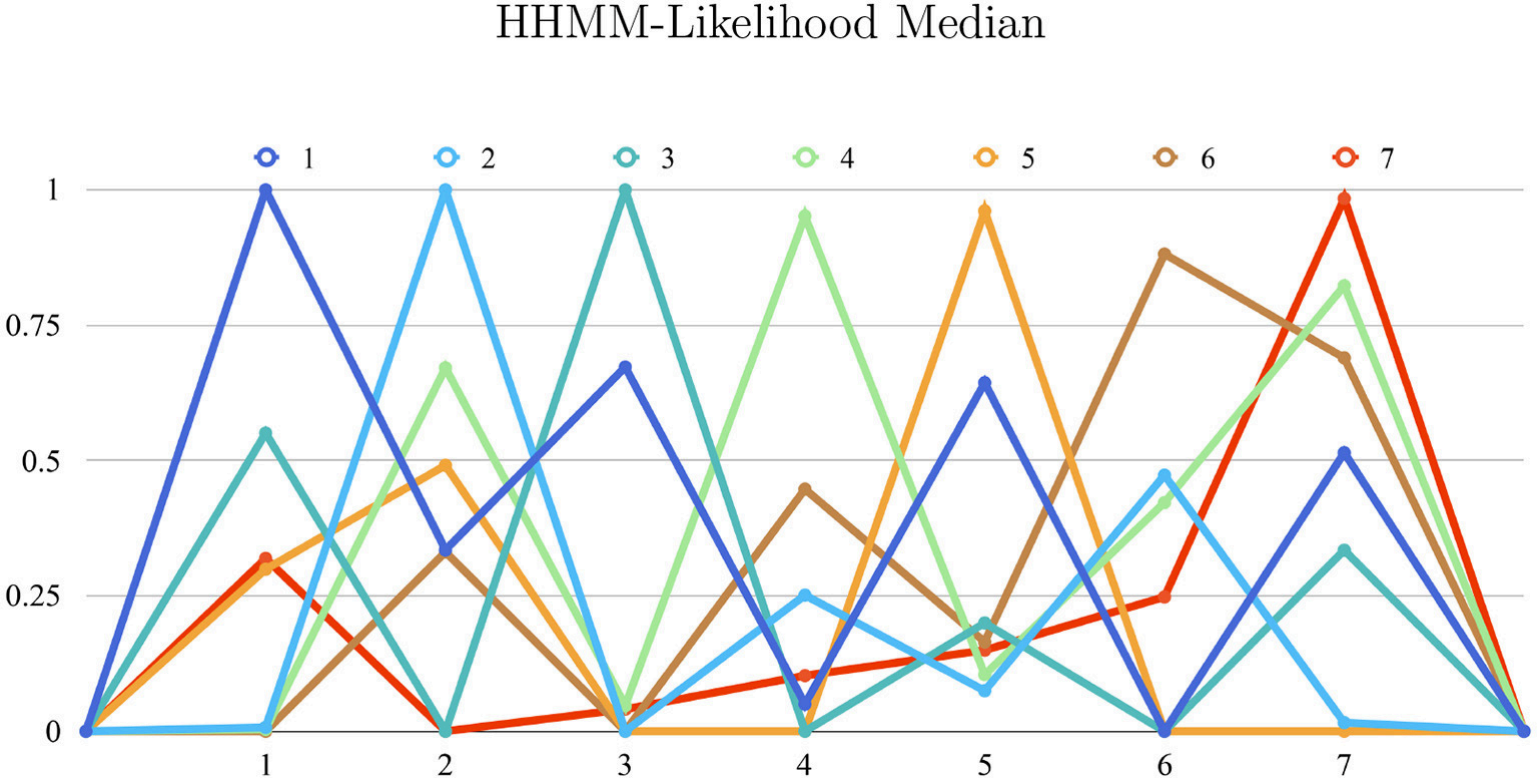
X-axis: gestures collection. Y-axis: 0. to 1. range as percentage of correct estimations (1:100). The graph shows a summarizing state of all gestures correct-estimations and their similitude. For instance, Gesture Détaché and Spiccato has some similarities in motion as they are closely described in the likelihood probability. Articulations: (1) Détaché, (2) Martelé, (3) Spiccato, (4) Ricochet, (5) Sautillé, (6) Staccato, (7) Bariolage.

We trained the HHMM previously described for real-time gesture estimation, resulting in a correctly classified instances percentage of 100% for detaché, martelé and spiccato; 95.1% for ricochet; 96.1% for sautillé; 88.1% for Staccato, and 98.4% for bariolage. These percentages represent the median of the gesture estimation in time. Each bow stroke has ten internal temporal states, and the model produces evaluations as likelihood probabilities progressions. The box-plot in the Figure 5 shows all HHMM-likelihood progression with 7846 samples, a mean of 42,394 samples per gesture. Similarly, Figure 6 shows The HHMM-likelihood median of the gesture recognition progression. Both figures give an insight of which gestures were better recorded and then described by the HHMM.

## 5. Discussion

**Figure 7:**
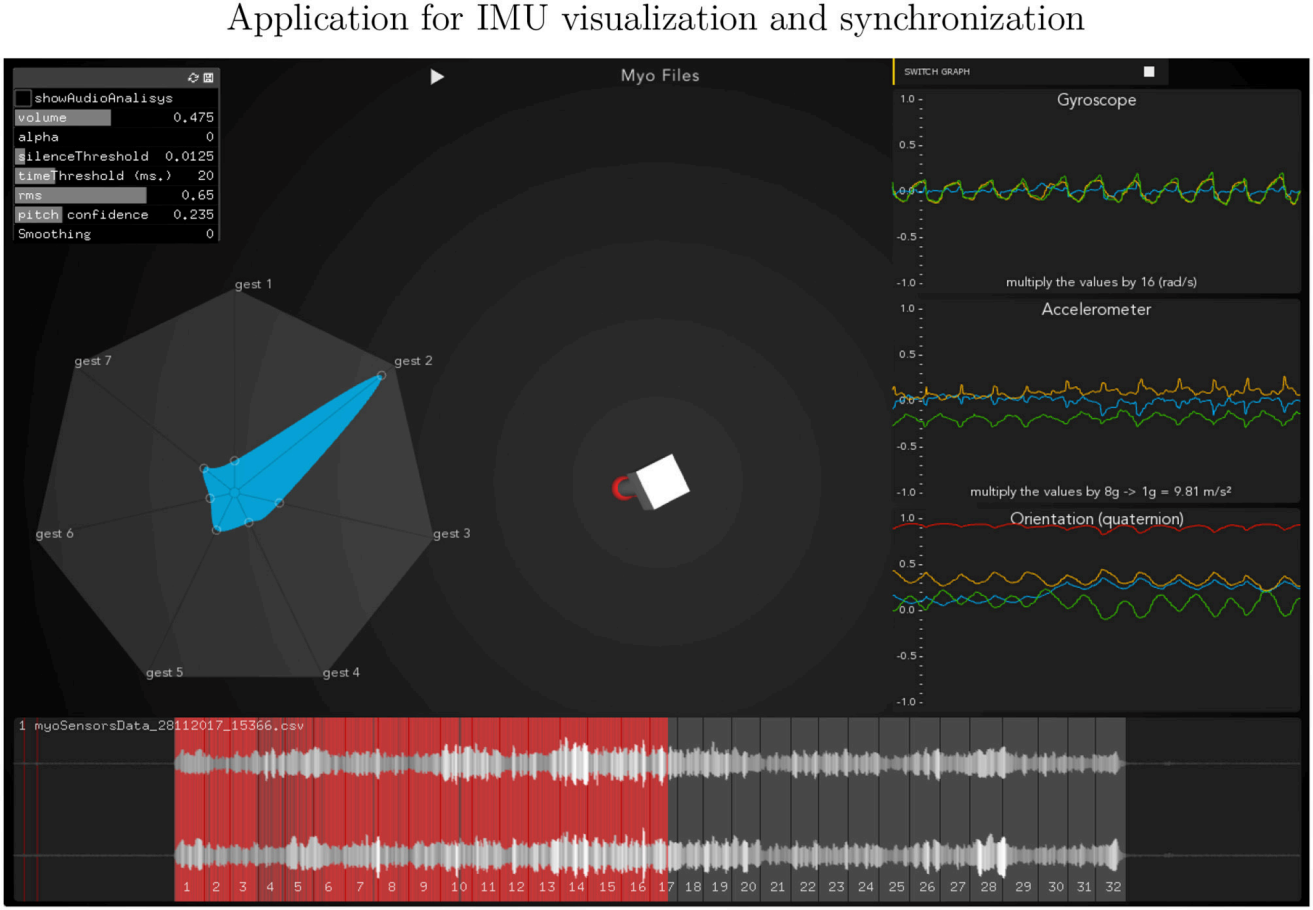
Openframework implementation to visualize and synchronize the IMU's and audio data. It reports in a spider chart the probability of the bow-stroke performed.

**Figure 8:**
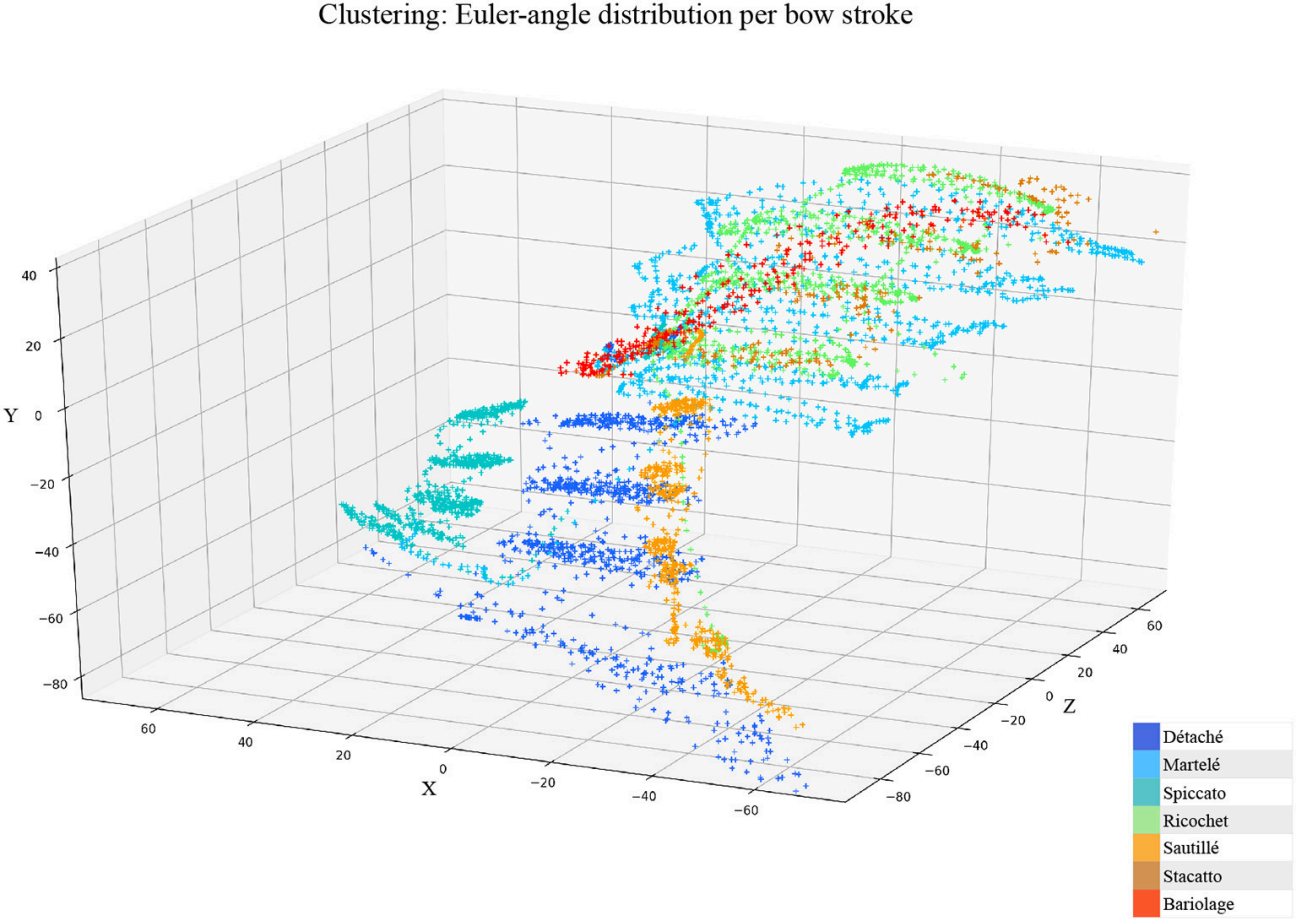
Cluster: Euler-angle spacial distribution of the seven articulations from the *Myo* device. Axis are estimated in centimeters.

**Figure 9:**
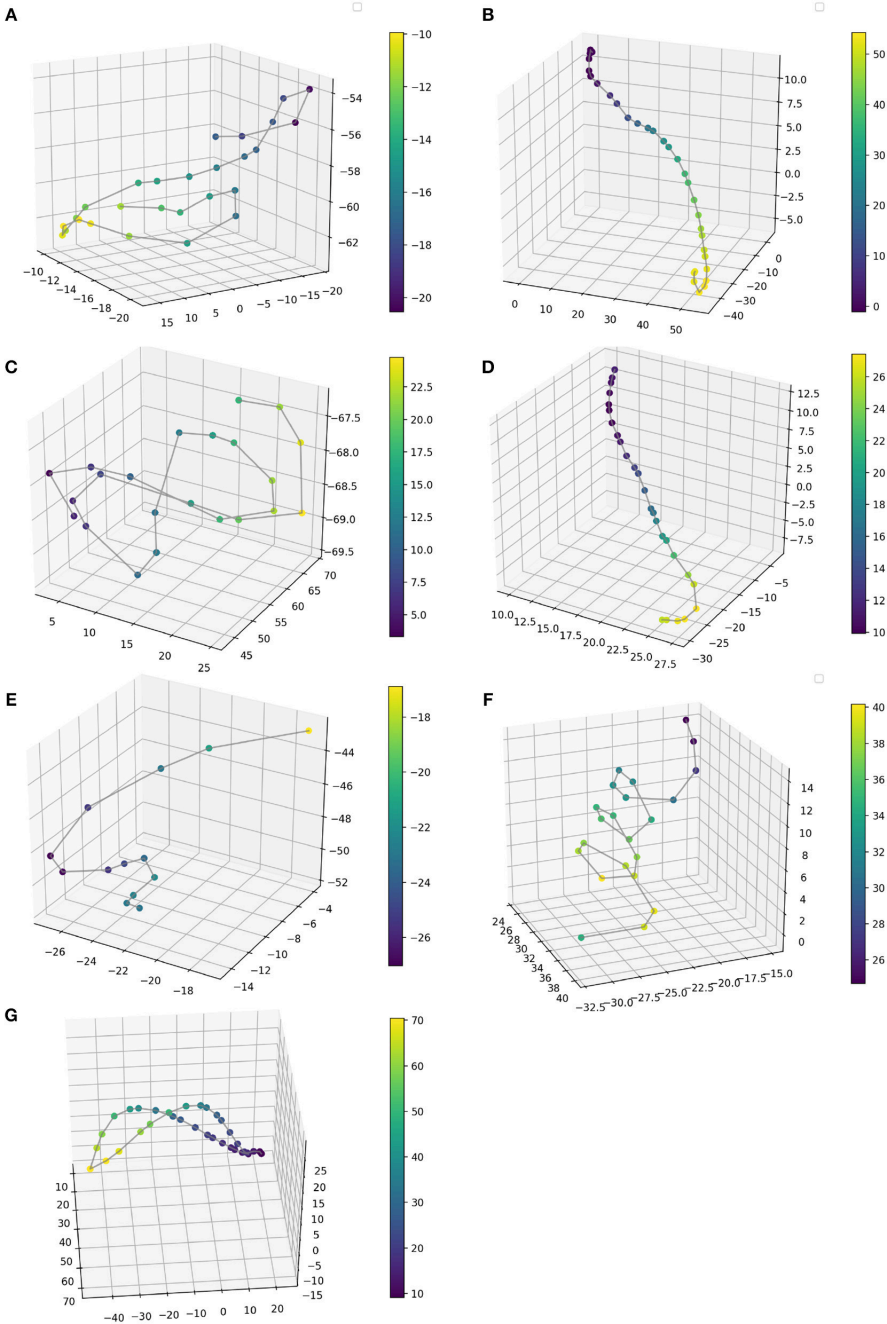
A single sample of the gestural phrase per each bow stroke technique. **(A)** Détaché, **(B)** Martelé, **(C)** Spiccato, **(D)** Ricochet, **(E)** Sautillé, **(F)** Staccato, **(G)** Bariolage. Color bar describes depth in z axis. Values are expressed in cm, taken from an positional origin first placed by the performer as a reference of the starting point, hence, values are the displacement from the original posture.

- **Gesture Prediction**: The HHMM has resulted in high accuracy when classifying the seven gestures given motion and audio data combined. It performs particularly well when it comes to classifying the détaché, martelé, spiccato gestures with 100% correct classification instances, and bariolage as well, with 98.4% of accuracy. Prediction of a gesture was made by computing the median of the ten states of each bow-stroke likelihood. Within a partial execution of a gesture, the model's estimation fluctuates due to the similarities between gestures and the partial information provided. In Figure 2 single bow strokes of each bow-technique are chosen to illustrate HHMM likelihood estimations. For instance, in the *Bariolage* graph (bottom left in the figure), a drop in likelihood estimation value halfway in the stroke can be seen, which is caused by the fact that the gesture has a fast bow swipe covering four strings in an ascending arpeggio followed by the inverse pattern in a descending arpeggio. The gesture of the arpeggio Bariolage is very similar as Détaché, a fact that is apparent at the end of the graph (280 ms) when the model's likelihood estimate of both gestures is practically the same. The choice of HHMM for training a gesture classifier was a natural one given that HHMM performs particularly well in problems where time plays an important role. Thus, as we mentioned before, not surprisingly they have been widely applied to problems in gesture recognition (Je et al., 2007; Caramiaux and Tanaka, 2013; Caramiaux et al., 2014; Françoise et al., 2014). Ricochet proved to be the most difficult gesture to be identified (i.e., produced the lowest accuracy) due to its similarity with Martelé. Both gestures are generated by a fast attack and then a smooth release, and they cover similar spacial areas (**Figure 9**). Ricochet sound is directly related to a wrist and finger technique applying a controlled weight over the strings causing a bouncing bow effect; for that reason, the audio descriptor helped to identify audible dissimilitudes among both gestures. A similar case is the case of Sautillé, which is produced with a fast attack motion, causing to be confused with Martelé. In an overall view, both datasets based on Myo and Myo + Audio descriptors reported very similar accuracy (93.32% and 94.62%), however, regarding bow-stroke recognition within the likelihood progression, audio descriptors increment the distance between similarities. For instance, *Martelé* and *Ricoche* has similar motion signatures (**Figure 9**) but the second gesture has a bouncing effect of the Bow over the violin's strings which is not reported in the IMU's data; hence, audio descriptor (RMS) gives the model the missing information.
- **Confusion Matrices**: Confusion Matrix of the Decision Tree (Table 2) reported high accuracy in the first five gestures and lower efficiency in the case of Staccato (f). Staccato gesture has a confusion of 9.5% with Ricochet, 9.2% with Martelé and 8.4% with Bariolage. Those gestures have some fundamental similarities, especially Martelé against Staccato, both start with a strong attack accent. For instance, in the Figures 5, 6, based on an HHMM-Likelihood Boxplots, those similarities are also expressed. In Staccato (f) case, other three gestures are present: Martelé (b), Ricochet (d) and Bariolage (g). It means that HHMM-likelihood was giving higher values in the temporal states to those gestures. The confusion matrix of the trained HHMM (Table 4) shows a correctly classified instances percentage of 100% for détaché, martelé and spiccato.
- **Pedagogical Application**: The ultimate purpose of the HHMM is to receive information in real-time about the progression of the bow stroke to give visual and haptic feedback to the students. To exemplify the idea, we have chosen randomly one single phrase within the seven different bow strokes. In Figure 2, the temporal progressions plotted are the seven bow strokes, where the x-axis is time (ms), and the y-axis is the probability estimation (scale 1:100). Likelihood estimation may be used to give real-time haptic feedback to a student to indicate deviations from the intended gesture. Such a feedback system is out of the scope of this paper and will be investigated in the future, including not only motion data but timing and pitch accuracy. In Figure 7 an OF application was designed for that purpose, a spider char gives information in real-time about the gestures recognition, as well it provides a visualization about Essentia descriptors and Myo data.
- **Myo Observations**: The Myo device needed to be placed carefully on the correct upward-front orientation. Changes in the disposition of the device in the forearm can cause deviation of the directional signals. For that reason, we focused on the “*mapping by demonstration”* principle (Françoise et al., 2014) where the models can be trained for particular users allowing in this way to tune the system for the master-apprentice scenario. In the Figure 8 a cluster of the seven gestures is plotted to give an insight into gestures different trajectories. It has to be noted that the data captured by the Myo does not precisely correspond to the bow motion. It is attached to the player's forearm and not the bow, thus not being able to capture the wrist movements. However, the results obtained show that the information captured by the Myo, i.e., forearm motion information, and the machine learning techniques applied, are sufficient to identify the singularities of the gestures studied. Figure 9 shows how each gesture has its particular spatial pattern and range of movement. In the figure, it is also possible to identify violin's string areas during the performance of the gestures.
- **Future Work**: We plan to explore deep learning models for the task to compare their accuracy against that of HHMMs. Another area of future research is to test the models in a real learning scenario: we plan to use the models to provide real-time feedback to violin students and compare learning outcomes in a group with feedback with a group with no-feedback. Deep Learning models were not implemented in this study as the dataset is limited in samples, we are planning to record several students and experts from Royal School of Music in London, performing those gestures to increment our data samples.

**Table 4:** Confusion matrix (HHMM).

**a**	**b**	**c**	**d**	**e**	**f**	**g**	**Class**
**1.000**	0.335	0.673	0.050	0.643	0.000	0.514	**a**
0.007	**1.000**	0.000	0.251	0.075	0.473	0.016	**b**
0.551	0.000	**1.000**	0.000	0.200	0.000	0.334	**c**
0.004	0.671	0.047	**0.951**	0.105	0.422	0.823	**d**
0.299	0.491	0.000	0.000	**0.961**	0.000	0.000	**f**
0.000	0.331	0.000	0.447	0.165	**0.881**	0.690	**g**
0.319	0.000	0.041	0.103	0.150	0.248	**0.984**	**h**

*Confusion matrix of the HHMM. Blue numbers are the correct predicted gestures as percentages of correct trials. Values are scaled from 0 to 1. Letters correspond to: (a) Détaché, (b) Martelé, (c) Spiccato, (d) Ricochet, (e) Sautillé, (f) Staccato, and (g) Bariolage*.

In TELMI project, colleges develop interactive applications to provide information to students about the quality of the sound and temporal precision of interpretation, in future work, we intend to embed the IMU's sensors into the Bow and Violin and merge both strategies, postural sensing technologies and a desktop/online app. Furthermore, we plan to implement the IMU device called *R-IOT* (Bitalino-IRCAM, 2018) with a size of 34 × 23 × 7 mm that can be incorporated into the Bow's frog, which will report gestural information in real-time in a similar manner of the *Myo*. It has a kit of Accelerometer, Gyroscope and Magnetometer, with a Sampling Rate: 200 Hz, Resolution: 16-bit (per IMU ch.), Communication: 2.4 GHz WiFi.

## Data Availability

The datasets [GENERATED/ANALYZED] for this study can be found in https://github.com/Dazzid/DataToRepovizz/tree/myo_to_repovizz/myo_recordings.

## Author Contributions

DD recorded, processed and analyzed the motion and audio data, and wrote the paper. RR supervised the methodology, the processing, and the analysis of the data, and contributed to the writing of the paper.

### Conflict of Interest Statement

The authors declare that the research was conducted in the absence of any commercial or financial relationships that could be construed as a potential conflict of interest.

## Footnotes

^1^MIT License. This gives everyone the freedoms to use OF in any context: commercial or non-commercial, public or private, open or closed source.
